# European Real-World Assessment of the Clinical Validity of a CE-IVD Panel for Ultra-Fast Next-Generation Sequencing in Solid Tumors

**DOI:** 10.3390/ijms241813788

**Published:** 2023-09-07

**Authors:** Nicola Normanno, José Carlos Machado, Edoardo Pescarmona, Simonetta Buglioni, Lara Navarro, Riziero Esposito Abate, Anabela Ferro, Rob Mensink, Matilde Lambiase, Virginie Lespinet-Fabre, Byron Calgua, Philip M. Jermann, Marius Ilié, Paul Hofman

**Affiliations:** 1 Cell Biology and Biotherapy Unit, Istituto Nazionale Tumori-IRCCS-Fondazione G. Pascale, 80131 Naples, Italy; nicnorm@yahoo.com (N.N.); r.espositoabate@istitutotumori.na.it (R.E.A.);; 2 Instituto de Investigação e Inovação em Saúde (i3S), University of Porto, 4200-135 Porto, Portugal; josem@ipatimup.pt (J.C.M.); aferro@ipatimup.pt (A.F.); rmensink@ipatimup.pt (R.M.); 3 Institute of Molecular Pathology and Immunology, University of Porto (Ipatimup), 4200-135 Porto, Portugal; 4 Department of Pathology, Faculty of Medicine, University of Porto (FMUP), 4200-319 Porto, Portugal; 5 I.R.C.C.S. Regina Elena National Cancer Institute, 00144 Rome, Italy; edoardo.pescarmona@ifo.it (E.P.); simonetta.buglioni@ifo.it (S.B.); 6 Consorcio Hospital General de Valencia, 46014 Valencia, Spain; lara.navarro@uv.es; 7 Laboratory of Clinical and Experimental Pathology, Biobank BB-0033-00025, FHU OncoAge, IHU RespirERA, CHU de Nice, Université Côte d’Azur, 06000 Nice, France; lespinet-fabre.v@chu-nice.fr (V.L.-F.); ilie.m@chu-nice.fr (M.I.); 8 Institute of Pathology, University Hospital Basel, 4031 Basel, Switzerland; byron.calgua@usb.ch (B.C.); philip.jermann@thermofisher.com (P.M.J.)

**Keywords:** CE-IVD, next-generation sequencing, biomarkers, molecular profiling, Oncomine Dx Express Test

## Abstract

Molecular profiling of solid tumors facilitates personalized, targeted therapeutic interventions. The ability to perform next-generation sequencing (NGS), especially from small tissue samples, in a short turnaround time (TAT) is essential to providing results that enable rapid clinical decisions. This multicenter study evaluated the performance of a CE in vitro diagnostic (IVD) assay, the Oncomine Dx Express Test, on the Ion Torrent Genexus System for detecting DNA and RNA variants in solid tumors. Eighty-two archived formalin-fixed paraffin embedded (FFPE) tissue samples from lung, colorectal, central nervous system, melanoma, breast, gastric, thyroid, and soft tissue cancers were used to assess the presence of single nucleotide variants (SNVs), insertions and deletions (indels), copy number variations (CNVs), gene fusions, and splice variants. These clinical samples were previously characterized at the various academic centers using orthogonal methods. The Oncomine Dx Express Test showed high performance with 100% concordance with previous characterization for SNVs, indels, CNVs, gene fusions, and splice variants. SNVs and indels with allele frequencies as low as 5% were correctly identified. The test detected all the expected *ALK*, *RET*, *NTRK1*, and *ROS1* fusion isoforms and *MET* exon 14-skipping splice variants. The average TAT from extracted nucleic acids to the final variant report was 18.3 h. The Oncomine Dx Express Test in combination with the Ion Torrent Genexus System is a CE-IVD-compliant, performant, and multicenter reproducible method for NGS detection of actionable biomarkers from a range of tumor samples, providing results in a short TAT that could support timely decision- making for targeted therapeutic interventions.

## 1. Introduction

The molecular profiling of solid tumors is mandatory for identifying actionable genomic alterations in a number of genes. The subsequent application of molecularly guided targeted therapies and personalized medicine improves patient response rates and prolongs survival for various cancers [1,2,3,4,5]. The National Comprehensive Cancer Network (NCCN) [6] and European Society for Medical Oncology (ESMO) [7] provide clinical practice guidelines in oncology, and in particular, the ESMO Scale for Clinical Actionability of Molecular Targets (ESCAT) classification provides a systematic framework to rank molecular targets based on clinical evidence of actionability. The ESCAT classification is a tier-based classification based on the level of evidence for the alteration–drug match [7]. ESCAT Tier I is the highest level of evidence indicating a list of ready-for-routine-use biomarkers that includes *EGFR*, *BRAF* V600E, and *MET* exon 14-skipping mutations and *ALK*, *ROS1*, *RET*, and *NTRK* fusions for non-small cell lung cancer (NSCLC); examples of other Tier I biomarkers are *ERBB2* amplifications, *PIK3CA* and *BRCA1/2* germline mutations, and microsatellite instability (MSI)-high for breast cancer and *IDH1* and *BRAF* mutations, MSI-high, human epidermal growth factor receptor 2 (HER2) overexpression, and *FGFR2* and *NTRK* fusions for cholangiocarcinoma [2,8].

Identifying the mutational profile of solid tumors by next-generation sequencing (NGS) offers unparalleled sensitivity and specificity for identifying individual mutations, enabling targeted treatment and patient-specific precision medicine. Recent advances have provided solutions to previous challenges related to low tumor cell percentage, small sample size, and long turnaround time (TAT) [9,10,11,12,13,14]. The Ion Torrent™ Genexus™ System (Thermo Fisher Scientific, Waltham, MA, USA) enables automation of targeted NGS workflows from library construction, can use genomic material from formalin-fixed paraffin-embedded (FFPE) tissue biopsies or cell-free total nucleic acids (cfTNA) from liquid biopsies, and provides results within 72 h [15]. In addition, when used in combination with the Oncomine Precision Assay, recent studies have demonstrated equal or superior performance to other NGS approaches for the detection of DNA and RNA variants in FFPE and liquid biopsy samples from a range of tumor types [1,16].

New EU legislation on in vitro diagnostic (IVD) medical devices, the In Vitro Diagnostic Regulation (IVDR) (EU) 2017/746, will be applied after a five-year transition period [16]. The new regulations aim to improve the quality, safety, and reliability of IVDs in countries that require CE-IVD-compliant products. Thus, both the assay used and the NGS system must be IVD-compliant. Under these regulations, IVD manufacturers and suppliers will be required to provide analytical performance data, scientific validity, peer-reviewed literature, and clinical performance data to demonstrate that their products meet these new, more stringent standards for clinical evidence. This will reduce variation and increase the consistency of in vitro diagnostics, which will have a positive impact on patient care and performance evaluation.

Here, we report a performance evaluation of the Oncomine Dx Express Test (ODxET, Thermo Fisher Scientific, Waltham, MA, USA), an IVD-compliant assay compatible with the Ion Torrent Genexus System. The ODxET is a pan-cancer panel that targets 46 genes such as *ALK*, *BRAF*, *EGFR*, *ERBB2*, *KRAS*, *MET*, *NTRK1/2/3*, *RET*, and *ROS1*, among others (Supplementary Table S1), covering 2769 unique variants, including hotspot mutations, copy number variations (CNVs), gene fusions, and splice variants corresponding to molecularly targeted drugs with labels, those in guidelines, as well as in global clinical trials [2,3]. In addition, 218 potential resistance-associated mutations across 22 genes are also included, providing a comprehensive assessment of tumor-associated genetic alterations. The ODxET panel includes other important emerging biomarkers for NSCLC, such as *STK11* and *KEAP1* (Supplementary Table S1). In combination with the Ion Torrent Genexus System, the ODxET requires only 10 ng of DNA and RNA extracted from two 5 µm FFPE slides with at least 10% tumor content, and results can be generated in as little as 24 h so that they can be integrated with immunohistochemistry results in one report. This efficient use of samples and fast TAT can also be applied to liquid biopsies.

This study reports the real-world evaluation of the ODxET panel in combination with the Ion Torrent Genexus System in six European academic research centers using pre-characterized FFPE samples from a range of tumor types and tumor cell percentages.

## 2. Results

### 2.1. Phase I: ODxET Performance Validation with Pre-Characterized TFS R&D Samples

Each site performed two runs using the commercially pre-characterized reference samples. One DNA analysis (Porto) and one RNA analysis (Nice) failed due to technical issues and were not included in the reproducibility analysis (Supplementary Table S2). However, both sequencing runs were completed on the system within the expected run time, and the corresponding RNA (Porto) and DNA (Nice) data from both runs could be evaluated. The total run time (Table 1), including library preparation, templating, sequencing, and analysis, was, on average, 18.3 h.

Overall, there was a high degree of agreement and consistency between the expected (TFS R&D) and observed (academic study center) readout measures for both DNA (allele frequency, CNV) and RNA variants (number of molecules) in the six samples (Table 2, Supplementary Table S2). Furthermore, a high degree of reproducibility was observed across the participating centers (Table 2).

### 2.2. Phase II: ODxET Performance Evaluation in Six Academic Centers

**Figure 1:**
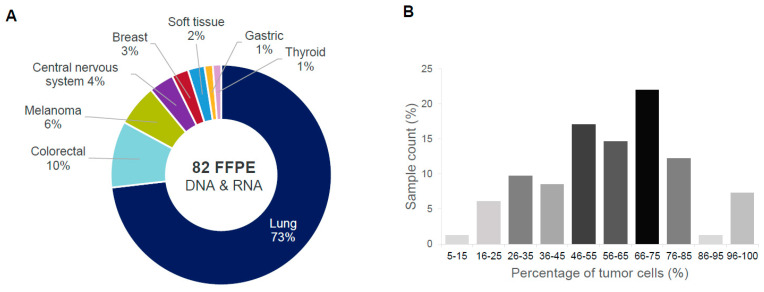
Cohort of pre-characterized FFPE samples used in the ODxET evaluation. (**A**) Tumor type distribution; (**B**) distribution of percentage of tumor cells in tumor tissues.

To assess analytical performance on real-life clinical samples, a total of 82 banked FFPE tissue samples were collected across the centers, including lung, colorectal, melanoma, central nervous system, breast, gastric, thyroid, and soft tissue cancers (Figure 1A). The tumor tissue ranged in tumor cell percentage from 5% to 100%, with the majority between 45 and 85% (Figure 1B). One RNA sample with an estimated tumor cellularity of 5% tumor cell percentage was included in this study despite the manufacturer-recommended minimum of 10% in order to maximize the number of fusion-positive samples in the cohort.

### 2.3. Detection of SNVs and Indels

**Figure 2:**
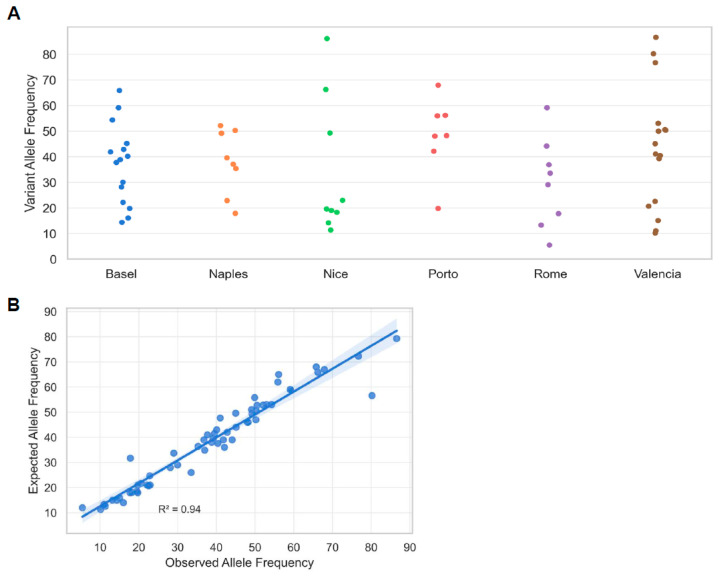
Range of allele frequencies detected using the ODxET. Each data point represents a mutation with ODxET and pre-characterization measurements of allele frequencies (alternate allele). (**A**) Allele frequencies were determined at each study center. (**B**) Correlation between expected allele frequency based on pre-characterization and ODxET observed allele frequency. Only mutations with pre-characterized testing information are shown.

The performance for detecting DNA variants (SNVs and indels) was evaluated across a set of 48 pre-characterized FFPE samples harboring a total of 63 known alterations in clinically relevant genes, including *EGFR*, *KRAS*, *TP53*, and *PIK3CA* (Supplementary Figure S2). Since pre-characterized samples were selected for this evaluation, the frequency of genes and variants does not necessarily reflect what is reported in the literature. All 63 known variants were detected with the ODxET assay. No additional clinically relevant variants were detected in this study as compared to the pre-characterization of these samples during routine testing. The allelic frequencies of the detected variants ranged from 5.4% to 86.6%, with an average of 38.6%, which was highly concordant with the frequencies observed with the corresponding orthogonal methods (R^2^ = 0.94, Figure 2A,B; Supplementary Table S3).

### 2.4. Assessment of Copy Number Variation

**Figure 3:**
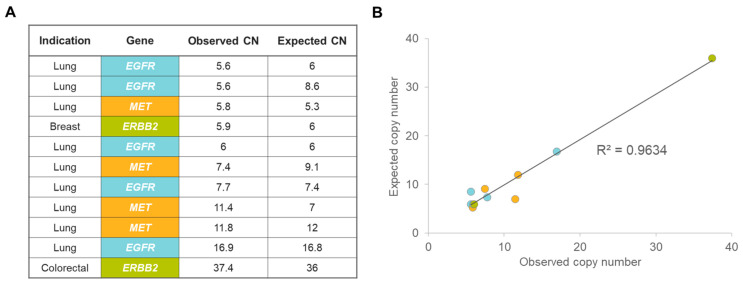
Assessment of copy number variation using ODxET. (**A**) Samples assessed with genes evaluated. CN: copy number. (**B**) Correlation between expected and observed copy numbers; each colored data point corresponds to the genes indicated in (**A**). Expected copy numbers were based on pre-characterization; observed copy numbers were based on ODxET evaluation. Only copy number amplifications with pre-characterized testing information are shown.

CNVs were determined across a set of 10 lung, breast, and colorectal cancer samples harboring a total of 11 known CNVs in the *EGFR*, *MET*, and *ERBB2* genes (Figure 3A) as determined by orthogonal methods, including the OPA, OFA, and INFORM HER2 FISH assays. The ODxET assay was able to detect all known CNVs, and the range of copy numbers detected was from 5.6 to 37.4, with a strong correlation (R^2^ = 0.96; Figure 3B) observed between the ODxET copy number measurement and the pre-characterized copy numbers.

### 2.5. Detection of Fusions and Splice Variants

**Figure 4:**
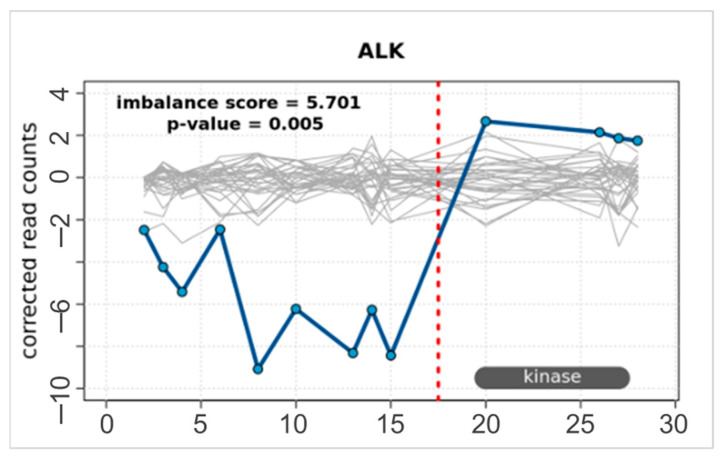
Detection of a novel ALK fusion. Exon-tiling imbalance in ODxET was able to detect a novel *ALK* fusion in a NSCLC sample pre-characterized as *ALK*-positive in Valencia using the Oncomine Precision Assay.

A total of 30 fusion isoforms and splice variants across 30 samples were included in the assessment of the ODxET assay, including the *ALK*, *RET*, *NTRK1*, and *ROS1* fusion isoforms and *MET* exon 14-skipping splice variants (Supplementary Figure S3). As for the other alterations described above, these samples had previously been characterized using the OPA, OFA, and OCAv3 panels, a customized Archer Fusion Plex assay, or FISH. All known fusions and splice variants were detected with the ODxET assay. In one NSCLC sample, an *ALK* fusion was detected only via the exon-tiling imbalance (Figure 4) (imbalance threshold score > 3.25, *p*-value < 0.05), as the specific fusion was not covered by targeted primers in the assay. The detection of fusions and splice variants by the ODxET assay was observed for a wide range of molecular counts (6–5023 molecules). For a number of variants, less than 50 molecules were detected, indicating good performance of the ODxET assay at low transcription levels (Supplementary Figure S4).

### 2.6. Analytical Performance of ODxET across Study Centers

The evaluation of the ODxET assay showed 100% concordance with the information from the orthogonal characterization across all tested variant types. Overall, excellent correlation with pre-characterization and high reproducibility between study centers were observed (Table 2). It is worth highlighting that the performance of the assay remained consistent regardless of whether manual or automated extraction and purification procedures were employed. This observation underscores the feasibility and validity of utilizing both manual and automated approaches for sample preparation aimed at sequencing via the ODxET method.

## 3. Discussion

The uptake of NGS in routine clinical diagnostics of solid tumors is still insufficient across Europe [17]. In fact, less than 10% of patients requiring molecular testing are evaluated with NGS-based molecular testing methods. Given the exponential increase in clinically significant genetic markers, there remains a high likelihood that important biomarkers will be missed [18]. The main reasons for the slow adoption of NGS include a lack of infrastructure and budget requirements, a lack of expertise, and a lack of implementation of NGS-based tests in the guidelines [17]. Therefore, the development of easy-to-use, rapid, automated NGS methods for detecting genetic alterations and mapping biomarkers for a number of solid tumors is of great value to routine clinical practice. Furthermore, the greatly improved TAT of these new technologies enables the timely delivery of genetic testing results to clinicians, enabling rapid decision-making and the implementation of personalized, biomarker-driven therapeutic interventions. Nevertheless, the lack of harmonized quality standards for tests currently developed in-house creates some uncertainty in test results. The new IVDR legislation aims to provide a harmonized regulatory framework to ensure the safety and performance of in vitro diagnostic medical devices on the European market [16]. To support the transition to the new regulation, a critical evaluation of existing CE-IVD-compliant NGS solutions that enable rapid TAT and the assessment of mutations in tumor driver genes for personalized therapies is required. Currently, the Ion Torrent Genexus System, an IVD device, provides a unique and automated end-to-end solution for NGS in solid tumors and in liquid biopsies in less than 24 h from nucleic acid availability. We recently reported that the Ion Torrent Genexus System, in combination with the OPA panel, enables the rapid and reproducible detection of DNA and RNA variants in a range of tumors and sample types with varying tumor cell content [16].

Here, we report that a CE-IVD-compliant assay version (the ODxET assay) is highly reproducible across six academic clinical and research centers and can detect both known and novel alterations, including key therapeutic targets within *EGFR*, *BRAF*, *KRAS*, *ALK*, *ROS1*, *NTRK*, *RET*, and other genes. Amplicon-based RNA sequencing can only detect known fusions. However, the availability of an exon-tiling assay also allows the detection of novel, unknown fusions that could eventually be confirmed using an orthogonal technique.

The ODxET combined with the Ion Torrent Genexus System produced results from nucleic acid to the final report within an average TAT of 18.3 h. A TAT longer than 14 days has been reported by many European centers performing NGS testing [17]. Long TATs are often not compatible with the clinical conditions of cancer patients who might be referred to generic chemotherapy in the absence of biomarker information in clinically meaningful time frames. The implications stemming from extended TATs have been thoroughly examined in a pair of recent studies [19,20]. These investigations have delved into the effects of long TATs, particularly within the context of patients diagnosed with NSCLC. The consensus drawn from these studies is that timely delivery of molecular testing results holds paramount importance, as it is a decisive factor in determining the optimal clinical outcome for patients. 

The ODxET requires very limited nucleic acid input. The ability to detect DNA and RNA genomic alterations with high sensitivity and specificity from small amounts of nucleic acid has significant advantages. First, it enables comprehensive, complete molecular profiling in situations where the amount of available biopsy material may be extremely limited, such as in many lung cancer biopsies, where cytological samples must be used [21]. This significantly limits the use of NGS technologies as well as single-analyte tests, which generally require larger amounts of material. Although the samples studied here were FFPE tumor tissues, the previous use of the OPA panel in combination with the Ion Torrent Genexus System to detect genetic alterations in liquid biopsies suggests that the ODxET assay will provide similar performance for variant detection in plasma-derived cell-free DNA [1].

Several other NGS systems are available and used worldwide [22]. The assay described in this study has several advantages from a clinical routine perspective, such as complete integration from library preparation to analysis in a single workflow, a lower TAT, an easy-to-use “plug-and-play” system that requires no prior bioinformatics experience, and integrated software for sequencing analysis and reporting, which make this system very easy to adopt in laboratories with little expertise in NGS.

Providing analytical performance data, scientific validity, and clinical performance data is critical to demonstrating the reliability of IVDs and showing that products meet rigorous standards for clinical application. Importantly, external validation, such as that demonstrated here by six academic clinical and research centers, increases the validity of new tests in a real-world setting, which is important not only for compliance with CE-IVD but also for patient care. The fact that the IVD-compliant ODxET assay has demonstrated high reproducibility with excellent sensitivity in several solid tumor types will enable the use of this assay for the identification of tumor mutation profiles and the rapid delivery of personalized medicine in compliance with EU regulations for in vitro diagnostics. In agreement with the recommendations from ESMO for NGS testing in clinical practice, this panel covers most level I alterations according to ESCAT. Larger panels for comprehensive genomic profiling should be used in academic centers that have access to clinical trials. Interestingly, recent data confirmed that the detection rate of ESCAT level I alterations is not different between small panels used in routine clinical testing and panels for comprehensive genomic profiling [23]. 

Within the scope of this study, it is important to acknowledge the limitations associated with the limited variety of orthogonal techniques. These methods, while distinct, employ mostly the same amplicon-based target-enrichment technology and sequencing platform as the method under validation. This parallel choice of technology might potentially lead to the introduction of shared systematic errors and biases that could influence the results. However, it is worth highlighting that all these selected orthogonal methodologies have undergone rigorous validation processes prior to our study. Specifically, these methods have been subject to thorough scrutiny and validation within the participating institutions. This collective experience and validation pedigree lend credence to the reliability of our comparisons despite the underlying technological commonality.

Excellent agreement was observed with previous characterizations of both DNA and RNA variants. High, reproducible performance was demonstrated across the six clinical and research centers, with an average TAT of 18.3 h. Thus, in combination with the Ion Torrent Genexus System, the ODxET enables rapid, sensitive, CE-IVD-compliant detection of clinically relevant biomarkers for personalized oncology.

## 4. Materials and Methods

### 4.1. Academic Clinical and Research Centers

This study was conducted in six European academic centers: the Institute of Medical Genetics and Pathology, University Hospital of Basel, Basel, Switzerland; the Istituto Nazionale Tumori “Fondazione Pascale” IRCCS, Naples, Italy; the Pasteur Hospital, University Côte d’Azur, Nice, France; the Institute of Molecular Pathology and Immunology of the University of Porto, Porto, Portugal; the I.R.C.C.S. Regina Elena National Cancer Institute, Rome, Italy; and the Consorcio Hospital General de Valencia, Valencia, Spain. Each study center profiled the same set of clinical samples pre-characterized by Thermo Fisher Scientific R&D (study phase I) and a selection from their own biobank of clinical samples characterized by gold standard and/or orthogonal methods (study phase II).

### 4.2. Clinical Samples

#### 4.2.1. Study Phase I: Pre-Characterized Samples

A set of six commercially pre-characterized samples was distributed among the centers to evaluate ODxET performance against the internal gold standard (Thermo Fisher Scientific (TFS) R&D). These samples included three SNV/indel (single nucleotide variants, insertions, and/or deletions) distinct variants (*EGFR* exon 19 deletion, *EGFR* exon 20 insertion, and *BRAF* V600E), one CNV (*ERBB2*), and two fusion variants (MET-MET.M13M15.1 and KIF5B-RET.K15R12.COSF1232.1) (Supplementary Table S2). The samples represented lung, bladder, and small intestinal cancers.

#### 4.2.2. Study Phase II: Biobank Clinical Samples

A total of 82 FFPE samples were collected across the centers. Inclusion criteria were availability of FFPE tissue, minimum tumor cellularity of 10%, and confirmed clinically relevant genetic alteration as determined by a validated orthogonal method. All centers used the same samples for DNA and RNA sequencing (Naples = 11, Nice = 11, Porto = 10, Rome = 12, and Valencia = 15), with the exception of Basel, which used a different set of samples for DNA and RNA sequencing (DNA = 12 and RNA = 11). The FFPE material used in this study had been pre-characterized using the Oncomine Precision Assay (OPA), Oncomine Focus Assay (OFA), Oncomine Comprehensive Assay v3 (OCAv3), Oncomine Comprehensive Assay Plus (OCA Plus), Oncomine Lung cfDNA Assay, Oncomine Solid Tumour DNA Kit (CE-IVD), Ion AmpliSeq Cancer Hotspot Panel v2, Custom Melanoma Panel (all assays Thermo Fisher Scientific, Waltham, MA, USA); Therascreen EGFR RGQ PCR Kit (Qiagen, Hilden, Germany); Custom Archer FusionPlex Assay (ArcherDx, Inc., Boulder, CO, USA); Cancer Panel DNA (Diatech Pharmocogenetics, Jesi, Italy); INFORM HER2 FISH (Roche, Basel, Switzerland); or Sanger Sequencing. A full list of orthogonal assays for each sample is available in Supplementary Table S3. Variants in all ESCAT Tiers for NSCLC were represented (Supplementary Figure S1), with other clinically relevant variants at ESCAT Tiers IA, IIB, and IIIA included for breast, colorectal, and gastric cancers.

### 4.3. Genomic Profiling by Next-Generation Sequencing

The ODxET was used to assess the presence of SNVs, indels, CNVs, gene fusions, and splice variants. For the detection of DNA and RNA variants in FFPE-derived material, 10 ng of starting material was used. In the Rome and Valencia centers, nucleic acid isolation, library preparation, templating, and sequencing were performed using the ODxET and the Genexus Purification System combined with the Genexus Integrated Sequencer, according to the manufacturer’s instructions. In the remaining centers, the Genexus Purification System was not available, and DNA and RNA were isolated sequentially from the tissue sections using the RecoverAll™ Total Nucleic Acid Isolation Kit for FFPE (Thermo Fisher Scientific, Waltham, MA, USA), according to the manufacturer’s instructions. Multiplexes of four samples were sequenced per lane using GX5 chips. NGS data analysis was performed using Genexus software version 6.6 (Thermo Fisher Scientific, Waltham, MA, USA) and a custom assay definition file to perform initial QC, including chip loading density, median read length, and number of mapped reads. It is noteworthy that the Genexus system performs all QC and data analysis steps fully automated, without any additional hands-on steps once sequencing is complete. This includes variant calling and optional report generation. Regarding QC, each run includes a positive control and a negative control. The positive control consists of 38 hotspot variants at defined variant allelic frequencies ranging from 3% to 21%, CNV controls for *ERBB2* and *MET*, and 17 fusion controls. Additionally, QC at the sample level includes a minimum average AQ20 read length of 64 bp and a maximum MAPD value of 0.5. Minimum allelic frequencies for SNV and indel detection are set at 2.5% and 2%, respectively. CNVs have a minimum copy number of 4. For RNA, the criteria for detecting fusions are either a minimum read count of 21 or a minimum molecular count of 3, with the exception of *ESR1-CCDC170* fusions and two *FGFR3-TACC3* fusions, where the thresholds are at 101 reads or 11 molecular counts. A total of four sequencing runs of the ODxET assay were carried out on the Ion Torrent Genexus System at each study center during the course of phases I and II.

### 4.4. Data Analysis

Data analysis and visualization were performed using Microsoft Office Excel software v.2208 (Microsoft Corporation., Redmond WA, USA) and GraphPad Prism v.9.4.1 (GraphPad Software, Boston, MA, USA). The correlation between variables was evaluated by calculating the R^2^ coefficient.

## 5. Conclusions

The adoption of NGS for the diagnosis of solid tumors in European clinical settings remains low, hindering the identification of important genetic markers. The reasons include infrastructure limitations, budget constraints, and a lack of expertise. The Ion Torrent Genexus System offers an easy-to-use, automated NGS solution with a rapid turnaround time. The CE-IVD-compliant ODxET assay, combined with this system, detects clinically relevant alterations in genes such as *EGFR*, *BRAF*, and *KRAS* within an average TAT of 18.3 h, starting from DNA and RNA. Rapid results are crucial for optimal patient care, especially in cancer cases. This IVD-compliant approach aids personalized oncology through the rapid identification of clinically relevant biomarkers.

## Figures and Tables

**Table 1:** Total run time for ODxET, including library preparation, templating, sequencing, and analysis for six samples and controls at each of the study centers.

Study Center	Total Run Time	
	Run #1 (h:min)	Run #2 (h:min)
Basel	18:06	18:03
Naples	18:22	18:15
Nice	18:00	17:56
Porto	18:16	18:01
Rome	18:38	18:34
Valencia	19:01	18:34

**Table 2:** Detection of expected DNA and RNA variants in pre-characterized samples by ODxET across study centers. TFS: Thermo Fisher Scientific; ^‡^ DNA sample failure—data from this run not included in final analysis.

	Sample	Cancer Type	Variant Type	Expected Variant (Pre-Characterized)	Unit of Measure	TFS R&D	Basel	Naples	Nice	Rome	Valencia
						Expected	Observed	Observed	Observed	Observed	Observed
DNA variants	1	Lung	Deletion	*EGFR* exon 19 del	Allele frequency	30.5%	31.4%	^‡^	34.6%	32.3%	29.8%
	2	Lung	Insertion	*EGFR* exon 20 ins	Allele frequency	41.4%	34.0%	36.9%	32.2%	36.7%	35.7%
	5	Bladder	CNV	*ERBB2* CNV	Copy number	35.3	37.5	36.9	37.7	36.0	36.3
	6	Small intestine	SNV	*BRAF* V600E	Allele frequency	53.3%	53.1%	51.5%	51.2%	53.3%	53.0%
RNA variants	3	Lung	Splice variant	*MET* Exon 14 Skip	No. of molecules	1787	1872	1955	515	1974	1889
	4	Lung	Fusion	*KIF5B-RET*	No. of molecules	110	124	47	134	84	143

## Data Availability

The raw data are available on demand to the corresponding author.

## Supplementary Materials

The following supporting information can be downloaded at: https://www.mdpi.com/article/10.3390/ijms241813788/s1.

## References

[1] Low S.K., Ariyasu R., Uchibori K., Hayashi R., Chan H.T., Chin Y.M., Akita T., Harutani Y., Kiritani A., Tsugitomi R. (2022). Rapid genomic profiling of circulating tumor DNA in non-small cell lung cancer using Oncomine Precision Assay with Genexus integrated sequencer. Transl. Lung Cancer Res..

[2] Mosele F., Remon J., Mateo J., Westphalen C.B., Barlesi F., Lolkema M.P., Normanno N., Scarpa A., Robson M., Meric-Bernstam F. (2020). Recommendations for the use of next-generation sequencing (NGS) for patients with metastatic cancers: A report from the ESMO Precision Medicine Working Group. Ann. Oncol..

[3] Pennell N.A., Mutebi A., Zhou Z.Y., Ricculli M.L., Tang W., Wang H., Guerin A., Arnhart T., Dalal A., Sasane M. (2019). Economic Impact of Next-Generation Sequencing versus Single-Gene Testing to Detect Genomic Alterations in Metastatic Non-Small-Cell Lung Cancer Using a Decision Analytic Model. JCO Precis. Oncol..

[4] Sheffield B.S., Beharry A., Diep J., Perdrizet K., Iafolla M.A.J., Raskin W., Dudani S., Brett M.A., Starova B., Olsen B. (2022). Point of Care Molecular Testing: Community-Based Rapid Next-Generation Sequencing to Support Cancer Care. Curr. Oncol..

[5] Zhong L., Li Y., Xiong L., Wang W., Wu M., Yuan T., Yang W., Tian C., Miao Z., Wang T. (2021). Small molecules in targeted cancer therapy: Advances, challenges, and future perspectives. Signal Transduct. Target. Ther..

[6] Ettinger D.S., Wood D.E., Aisner D.L., Akerley W., Bauman J.R., Bharat A., Bruno D.S., Chang J.Y., Chirieac L.R., D’Amico T.A. (2022). Non-Small Cell Lung Cancer, Version 3.2022, NCCN Clinical Practice Guidelines in Oncology. J. Natl. Compr. Cancer Netw..

[7] Mateo J., Chakravarty D., Dienstmann R., Jezdic S., Gonzalez-Perez A., Lopez-Bigas N., Ng C.K.Y., Bedard P.L., Tortora G., Douillard J.Y. (2018). A framework to rank genomic alterations as targets for cancer precision medicine: The ESMO Scale for Clinical Actionability of molecular Targets (ESCAT). Ann. Oncol..

[8] Vogel A., Bridgewater J., Edeline J., Kelley R.K., Klumpen H.J., Malka D., Primrose J.N., Rimassa L., Stenzinger A., Valle J.W. (2022). Biliary tract cancer: ESMO Clinical Practice Guideline for diagnosis, treatment and follow-up. Ann. Oncol..

[9] Coghlin C.L., Smith L.J., Bakar S., Stewart K.N., Devereux G.S., Nicolson M.C., Kerr K.M. (2010). Quantitative analysis of tumor in bronchial biopsy specimens. J. Thorac. Oncol..

[10] Hayashi H., Tanishima S., Fujii K., Mori R., Okada C., Yanagita E., Shibata Y., Matsuoka R., Amano T., Yamada T. (2020). Clinical impact of a cancer genomic profiling test using an in-house comprehensive targeted sequencing system. Cancer Sci..

[11] Heitzer E., Haque I.S., Roberts C.E.S., Speicher M.R. (2019). Current and future perspectives of liquid biopsies in genomics-driven oncology. Nat. Rev. Genet..

[12] Kou T., Kanai M., Yamamoto Y., Kamada M., Nakatsui M., Sakuma T., Mochizuki H., Hiroshima A., Sugiyama A., Nakamura E. (2017). Clinical sequencing using a next-generation sequencing-based multiplex gene assay in patients with advanced solid tumors. Cancer Sci..

[13] Mileham K.F., Schenkel C., Bruinooge S.S., Freeman-Daily J., Basu Roy U., Moore A., Smith R.A., Garrett-Mayer E., Rosenthal L., Garon E.B. (2022). Defining comprehensive biomarker-related testing and treatment practices for advanced non-small-cell lung cancer: Results of a survey of U.S. oncologists. Cancer Med..

[14] Benson A.B., Venook A.P., Cederquist L., Chan E., Chen Y.J., Cooper H.S., Deming D., Engstrom P.F., Enzinger P.C., Fichera A. (2017). Colon Cancer, Version 1.2017, NCCN Clinical Practice Guidelines in Oncology. J. Natl. Compr. Cancer Netw..

[15] Ilié M., Hofman V., Bontoux C., Heeke S., Lespinet-Fabre V., Bordone O., Lassalle S., Lalvée S., Tanga V., Allegra M. (2022). Setting Up an Ultra-Fast Next-Generation Sequencing Approach as Reflex Testing at Diagnosis of Non-Squamous Non-Small Cell Lung Cancer; Experience of a Single Center (LPCE, Nice, France). Cancers.

[16] mdi Europa In Vitro Diagnostics EU Directive IVDR—In Vitro Diagnostic Medical Devices Regulation (EU) 2017/746. https://mdi-europa.com/ivdr-in-vitro-diagnostic-medical-devices-regulation-eu-2017-746/.

[17] Bayle A., Bonastre J., Chaltiel D., Latino N., Rouleau E., Peters S., Galotti M., Bricalli G., Besse B., Giuliani R. (2023). ESMO study on the availability and accessibility of biomolecular technologies in oncology in Europe. Ann. Oncol..

[18] Mateo J., Steuten L., Aftimos P., Andre F., Davies M., Garralda E., Geissler J., Husereau D., Martinez-Lopez I., Normanno N. (2022). Delivering precision oncology to patients with cancer. Nat. Med..

[19] Aggarwal C., Marmarelis M.E., Hwang W.T., Scholes D.G., McWilliams T.L., Singh A.P., Sun L., Kosteva J., Costello M.R., Cohen R.B. (2023). Association between Availability of Molecular Genotyping Results and Overall Survival in Patients with Advanced Nonsquamous Non-Small-Cell Lung Cancer. JCO Precis. Oncol..

[20] Scott J.A., Lennerz J., Johnson M.L., Gordan L.N., Dumanois R.H., Quagliata L., Ritterhouse L.L., Cappuzzo F., Wang B., Xue M. (2023). Compromised Outcomes in Stage IV Non-Small-Cell Lung Cancer with Actionable Mutations Initially Treated without Tyrosine Kinase Inhibitors: A Retrospective Analysis of Real-World Data. JCO Oncol. Pract..

[21] Bellevicine C., Malapelle U., Vigliar E., Pisapia P., Vita G., Troncone G. (2017). How to prepare cytological samples for molecular testing. J. Clin. Pathol..

[22] Cainap C., Balacescu O., Cainap S.S., Pop L.A. (2021). Next Generation Sequencing Technology in Lung Cancer Diagnosis. Biology.

[23] Dalens L., Niogret J., Kaderbhai C.G., Boidot R. (2022). Is There a Role for Large Exome Sequencing in the Management of Metastatic Non-Small Cell Lung Cancer: A Brief Report of Real Life. Front. Oncol..

