# Supplementary material for: European Real-World Assessment of the Clinical Validity of a CE-IVD Panel for Ultra-Fast Next-Generation Sequencing in Solid Tumors
Source: Int J Mol Sci. 2023 Sep 7;24(18):13788. doi: 10.3390/ijms241813788 (PMC10531166; doi:10.3390/ijms241813788)
Supplement: Supplementary file 1 [file ijms-24-13788-s001.zip › ijms-2565357-supplementary.pptx]

## Slide 1
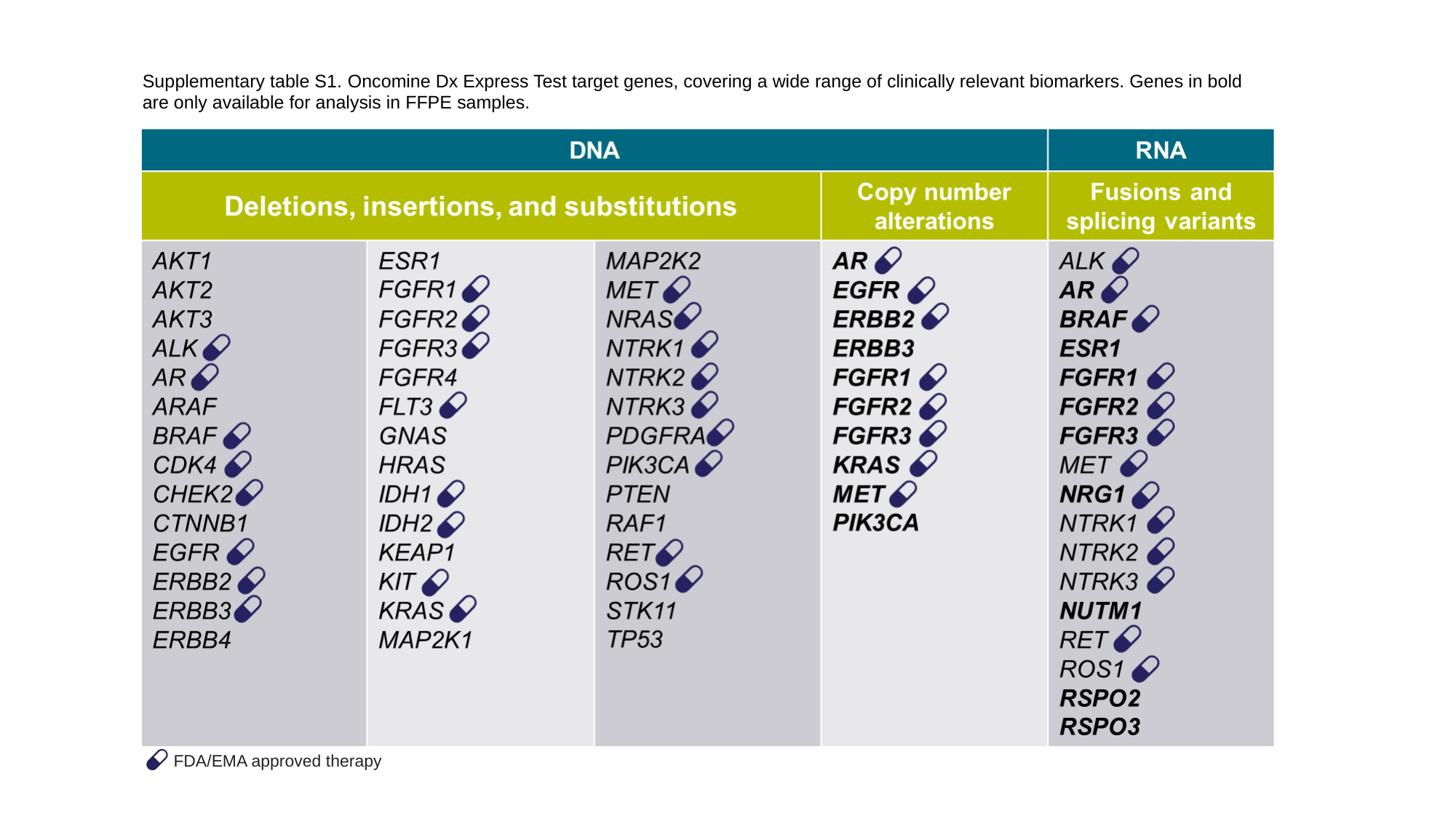

Supplementary table S1. Oncomine Dx Express Test target genes, covering a wide range of clinically relevant biomarkers. Genes in bold are only available for analysis in FFPE samples.
FDA/EMA approved therapy

## Slide 2
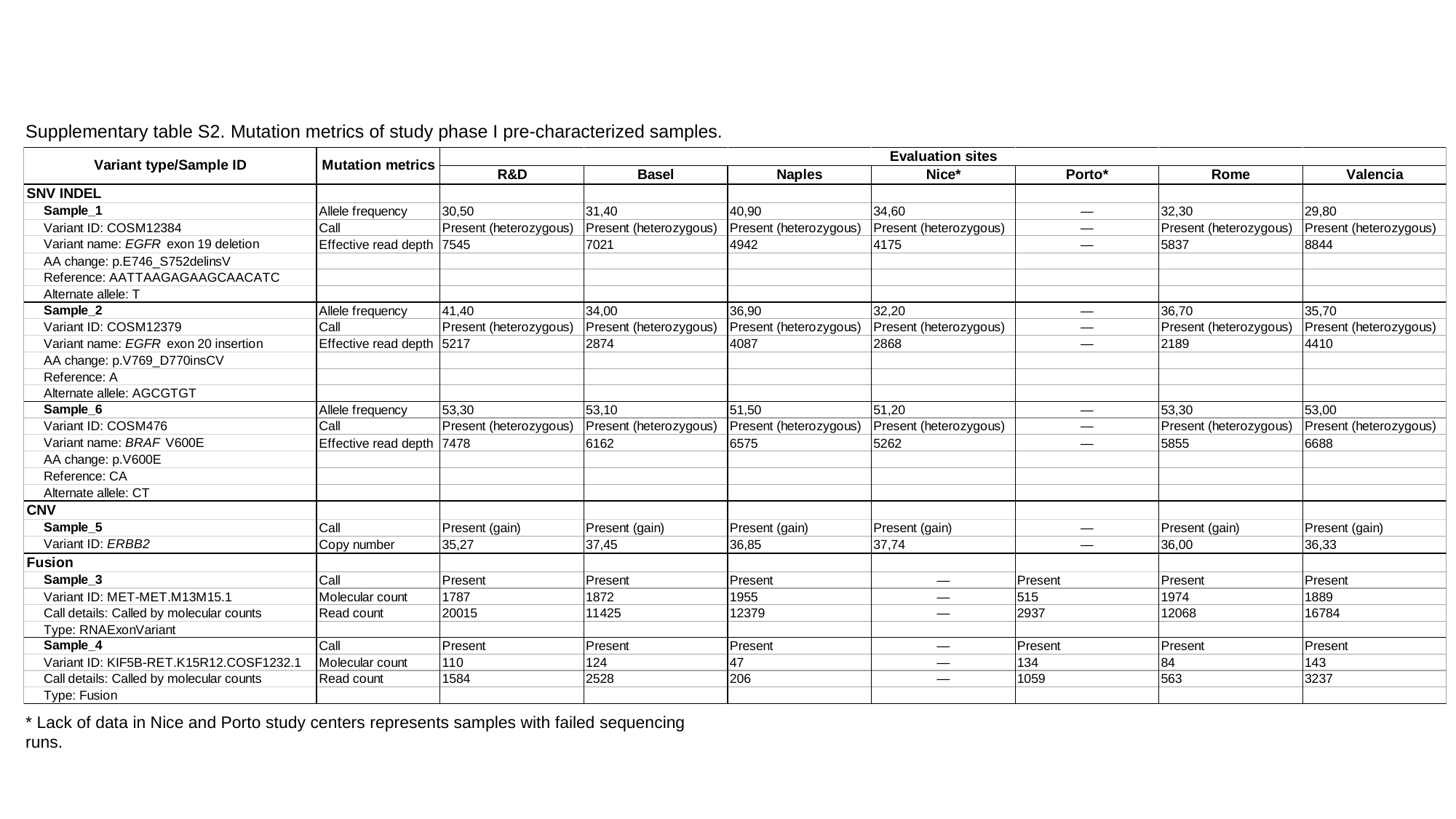

Supplementary table S2. Mutation metrics of study phase I pre-characterized samples.
* Lack of data in Nice and Porto study centers represents samples with failed sequencing runs.

## Slide 3
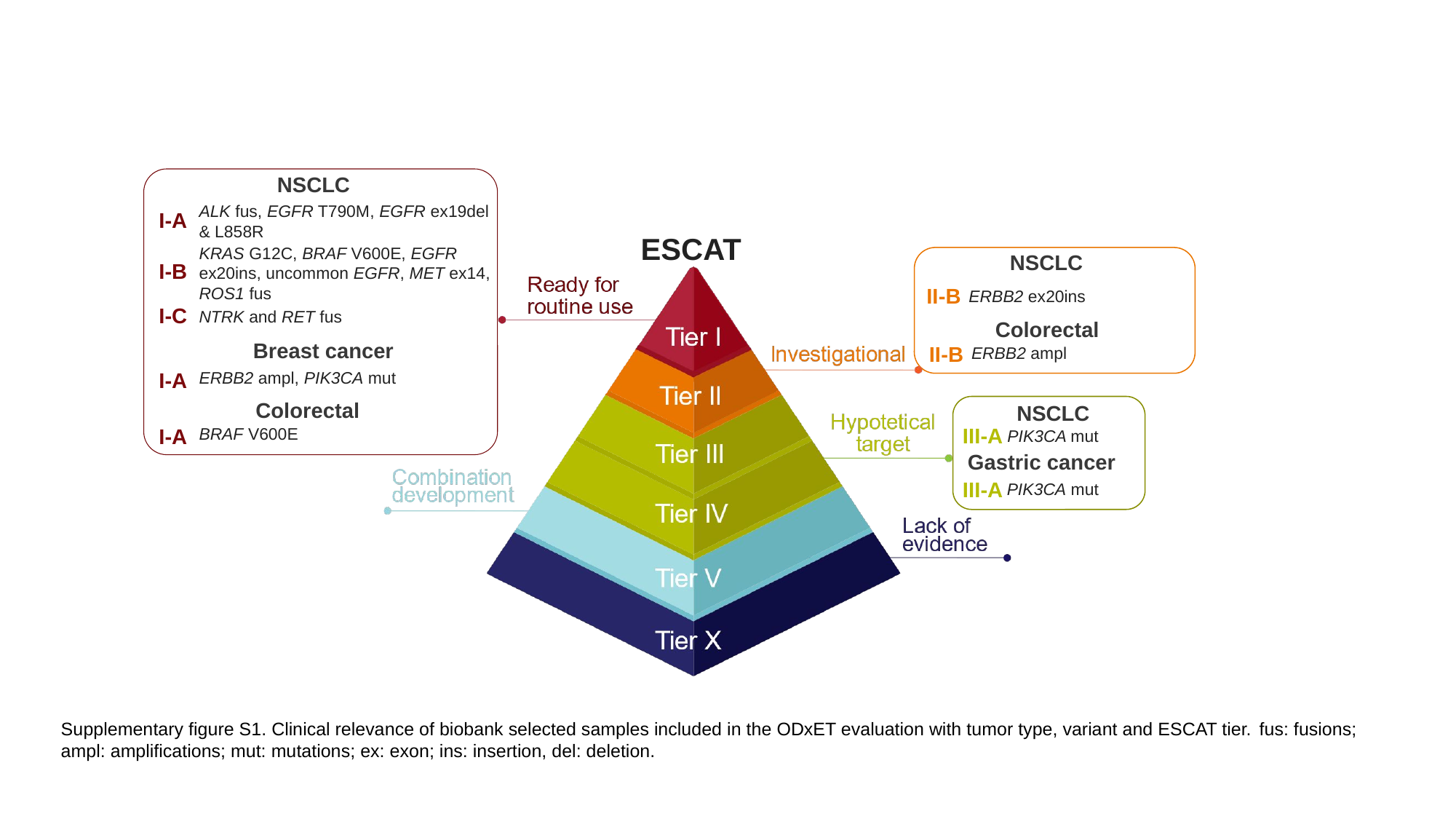

NSCLC
I-A
ALK fus, EGFR T790M, EGFR ex19del & L858R
ESCAT
KRAS G12C, BRAF V600E, EGFR ex20ins, uncommon EGFR, MET ex14, ROS1 fus
NSCLC
II-B
ERBB2 ex20ins
Colorectal
II-B
ERBB2 ampl
I-B
I-C
NTRK and RET fus
Breast cancer
ERBB2 ampl, PIK3CA mut
I-A
Colorectal
NSCLC
III-A
PIK3CA mut
Gastric cancer
III-A
PIK3CA mut
BRAF V600E
I-A
Supplementary figure S1. Clinical relevance of biobank selected samples included in the ODxET evaluation with tumor type, variant and ESCAT tier. fus: fusions; ampl: amplifications; mut: mutations; ex: exon; ins: insertion, del: deletion.

## Slide 4
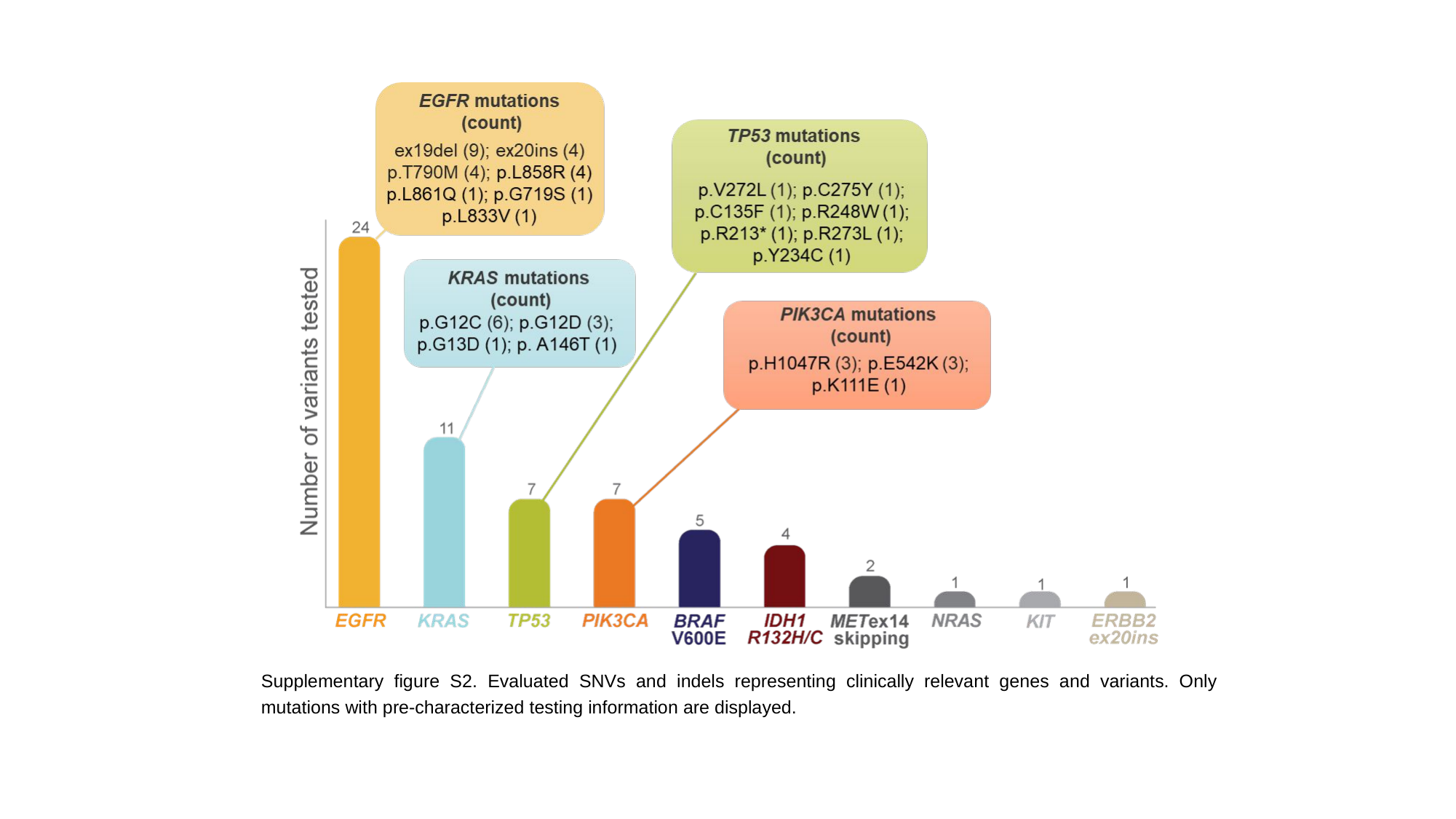

Supplementary figure S2. Evaluated SNVs and indels representing clinically relevant genes and variants. Only mutations with pre-characterized testing information are displayed.

## Slide 5
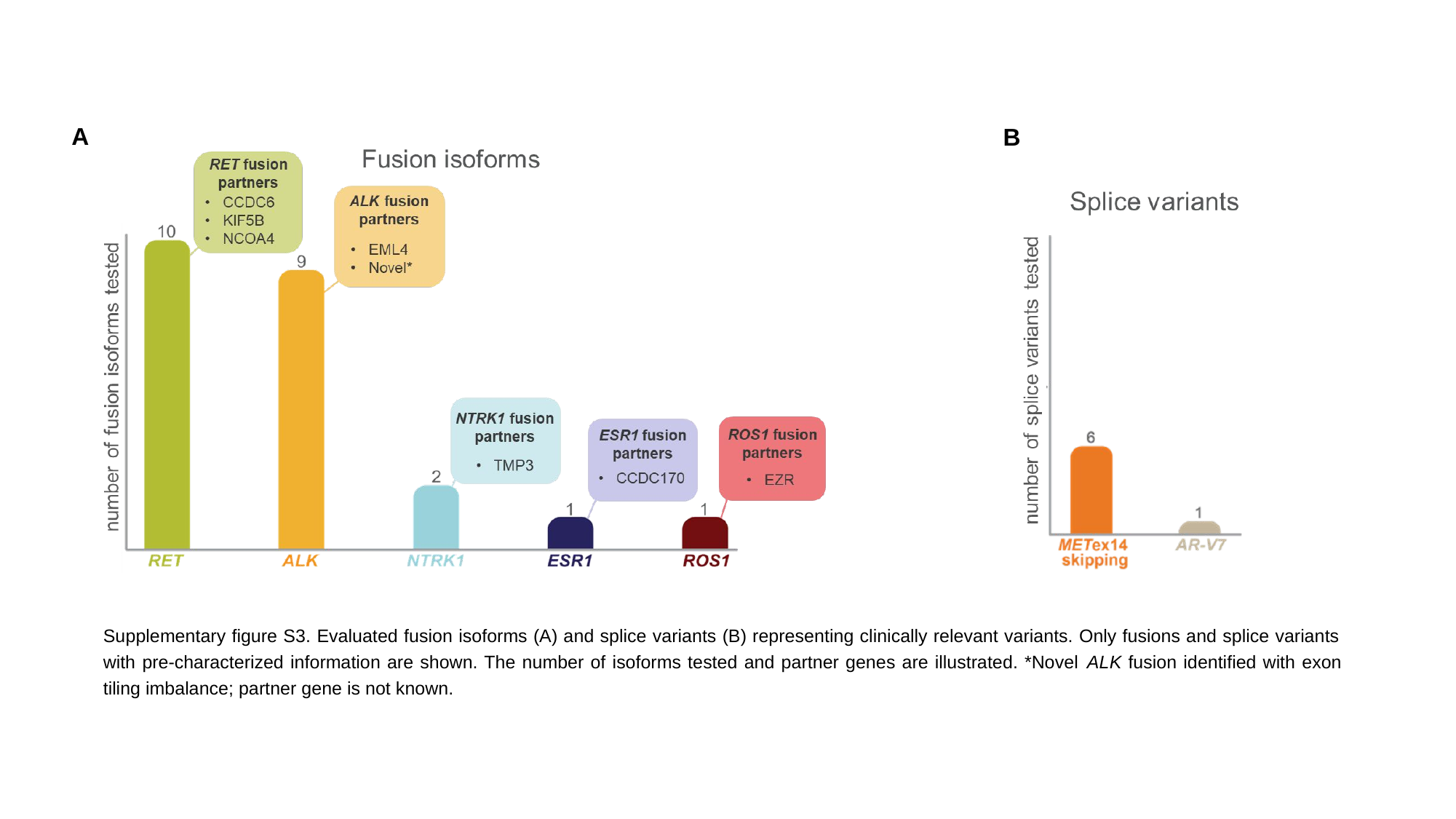

A
B
Supplementary figure S3. Evaluated fusion isoforms (A) and splice variants (B) representing clinically relevant variants. Only fusions and splice variants with pre-characterized information are shown. The number of isoforms tested and partner genes are illustrated. *Novel ALK fusion identified with exon tiling imbalance; partner gene is not known.

## Slide 6
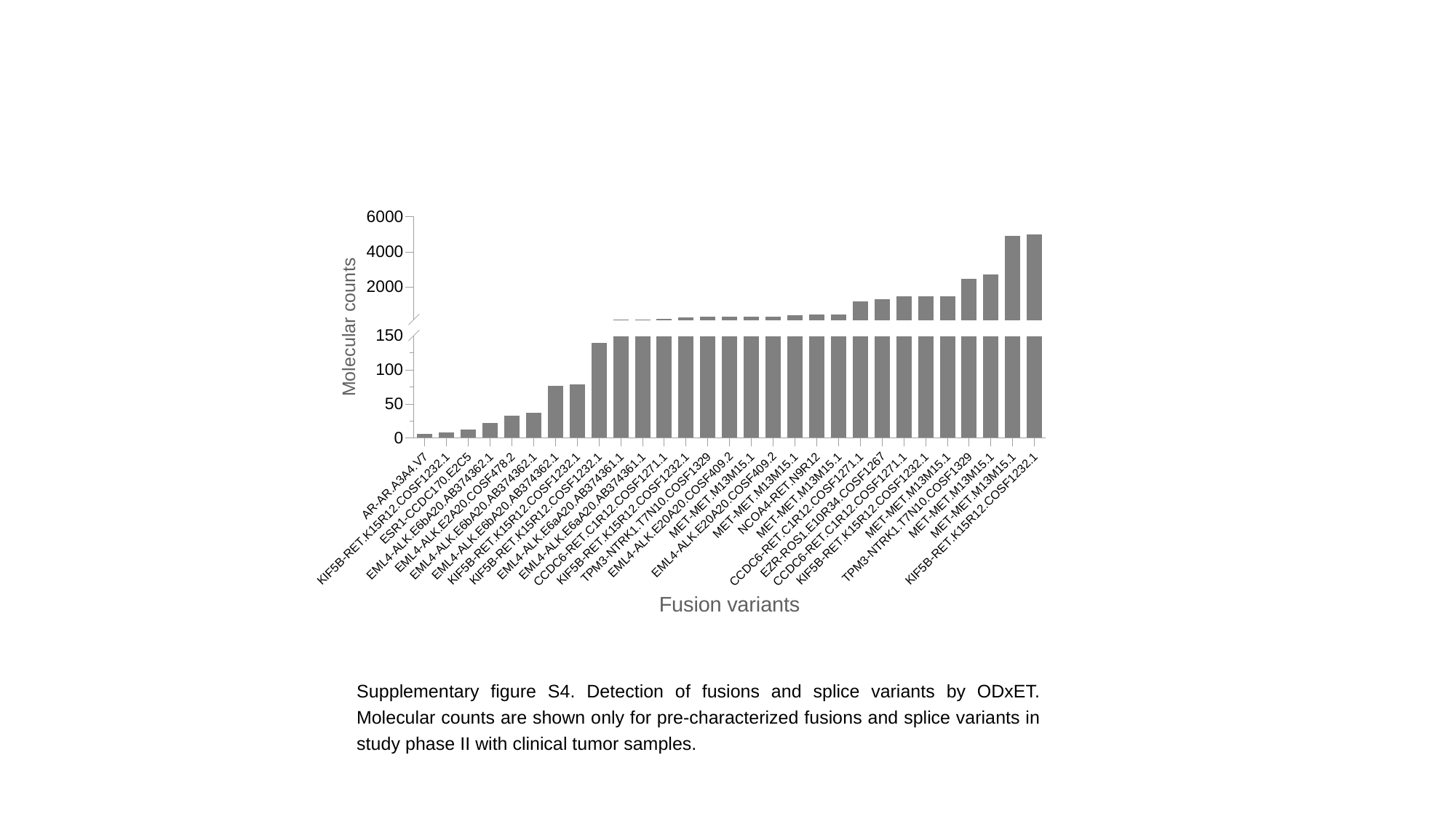

Supplementary figure S4. Detection of fusions and splice variants by ODxET. Molecular counts are shown only for pre-characterized fusions and splice variants in study phase II with clinical tumor samples.
